# Reduced ratio of eicosapentaenoic acid and docosahexaenoic acid to arachidonic acid is associated with early onset of acute coronary syndrome

**DOI:** 10.1186/s12937-015-0102-4

**Published:** 2015-10-29

**Authors:** Shusuke Yagi, Ken-ichi Aihara, Daiju Fukuda, Akira Takashima, Mika Bando, Tomoya Hara, Sachiko Nishimoto, Takayuki Ise, Kenya Kusunose, Koji Yamaguchi, Takeshi Tobiume, Takashi Iwase, Hirotsugu Yamada, Takeshi Soeki, Tetsuzo Wakatsuki, Michio Shimabukuro, Masashi Akaike, Masataka Sata

**Affiliations:** 1 Department of Cardiovascular Medicine, Institute of Biomedical Sciences, Tokushima University Graduate School, 3-18-15 Kuramoto, Tokushima, 770-8503 Japan; 2 Department of Hematology, Endocrinology and Metabolism, Institute of Biomedical Sciences, Tokushima University Graduate School, 3-18-15 Kuramoto, Tokushima, 770-8503 Japan; 3 Department of Nutrition and Metabolism, Institute of Biomedical Sciences, Tokushima University Graduate School, 3-18-15 Kuramoto, Tokushima, 770-8503 Japan; 4 Department of Cardio-Diabetes Medicine, Institute of Biomedical Sciences, Tokushima University Graduate School, 3-18-15 Kuramoto, Tokushima, 770-8503 Japan; 5 Department of Medical Education, Institute of Biomedical Sciences, Tokushima University Graduate School, 3-18-15 Kuramoto, Tokushima, 770-8503 Japan

**Keywords:** Polyunsaturated fatty acids (PUFA), Eicosapentaenoic acid (EPA), Docosahexaenoic acid (DHA), Acute coronary syndrome (ACS)

## Abstract

**Background:**

The hospitalization rate for acute coronary syndrome (ACS) for people aged ≤50 has remained stable over the past decade. Increased serum levels of n-3 polyunsaturated fatty acids (PUFAs), such as eicosapentaenoic acid (EPA) and docosahexaenoic acid (DHA), are associated with a decreased incidence of cardiovascular events and mortality in older patients; however, it is currently unknown whether reduced serum levels of n-3 PUFAs is also a risk factor for ACS in patients aged ≤50 years.

**Methods and results:**

We retrospectively reviewed 102 (male/ female 73/29) Japanese ACS patients whose serum levels of EPA/arachidonic acid (AA) and DHA/AA were evaluated on admission. The EPA/AA ratio was the lowest in patients aged ≤50 compared to patients aged 51–74 and ≥75. Pearson correlation analysis showed that early ACS onset was associated with low EPA/AA and DHA/AA ratios, and multiple regression analysis determined that decreased ratios of EPA/AA and DHA/AA, and male sex, current smoker status, increased body mass index and triglyceride levels, independently correlated with early ACS onset. Conversely, low-density and high-density lipoproteins, glycated hemoglobin, and hypertension did not correlate with early ACS onset. Subgroup analyses of male patients revealed that decreased ratios of EPA/AA and DHA/AA independently correlated with early ACS onset.

**Conclusion:**

Decreased EPA/AA and DHA/AA ratios may be risk factors for early onset of ACS, suggesting that reduced EPA/AA and DHA/AA may represent targets for preventing ACS in Japanese young people.

**Electronic supplementary material:**

The online version of this article (doi:10.1186/s12937-015-0102-4) contains supplementary material, which is available to authorized users.

## Background

In relation to the increased numbers of elderly people, the rate of young patients with acute coronary syndrome (ACS), including acute myocardial infarction (AMI), in Japan has been relatively decreasing; however, the age-adjusted incidence of AMI over the last 30 years has conversely been increasing, especially in men [1]. In addition, the clinical features and outcomes of ACS in young subjects (≤45 years) are considered to differ from those in non-young subjects (>45 years), owing to differences in the lifestyle and diet patterns [2]. Therefore, identification of new life-style related risk factors for ACS is important, especially in young subjects. It has been reported that reduced serum levels of n-3 polyunsaturated fatty acids (PUFAs), including eicosapentaenoic acid (EPA) and docosahexaenoic acid (DHA), are associated with an increased incidence of cardiovascular events and mortality [3, 4]. Additionally, a decreased ratio of serum EPA/arachidonic acid (AA) has been demonstrated to significantly correlate with the onset of ACS [5], and the serum EPA/AA ratio has been shown to stepwise increase with age [6], suggesting that reduced serum levels of n-3 PUFAs may represent a risk factor for ACS, especially in young subjects. However, it is currently unknown whether reduced serum levels of n-3 PUFAs is a risk factor for early onset ACS, and, accordingly, this study aimed to investigate this issue.

## Material and methods

### Patients and study design

We retrospectively reviewed 102 consecutive Japanese patients diagnosed with ACS who underwent emergent percutaneous coronary intervention in the Department of Cardiovascular Medicine at Tokushima University Hospital between January 2009 and June 2014. The patients were stratified into 3 groups according to age (≤50, 51–74, and ≥75 years) and analyzed.

Because all patients aged ≤50 were male, we moreover performed subgroup analyses after excluding all female patients, resulting in a total of 73 male patients out of the total 102 ACS patients being included in the analyses.

ACS included AMI and unstable angina. AMI was defined as a transient increase of the MB fraction of creatine kinase to a threshold of 3 times the 99th percentile of the upper reference limit (150 U/ L) after percutaneous coronary intervention in patients with ischemic symptoms and/or typical electrocardiographic findings (ST elevation) [7]. Unstable angina was defined as angina at rest, accelerated exertional angina combined with typical electrocardiographic changes (ST depression), or an increase in the intensity of anti-ischemic therapy with a transient increase of the MB fraction of creatine kinase to a threshold of less than 3 times the 99th percentile of the upper reference limit, as described previously [5]. The exclusion criteria were as follows: use of fish oil supplements or n-3 fatty acid-containing drugs. In addition, patients with symptomatic, active malignant diseases or liver dysfunction (aspartate aminotransferase levels >100 IU/L, alanine aminotransferase levels >100 IU/L) were also excluded.

Hypertensive patients were defined as those with a systolic blood pressure ≥140 mmHg and/or diastolic blood pressure ≥90 mmHg, and/or individuals receiving antihypertensive medications. Dyslipidemic patients were defined as those with a low-density lipoprotein cholesterol (LDL-C) level ≥140 mg/dL, a triglyceride level ≥150 mg/dL, a high-density lipoprotein cholesterol (HDL-C) level <40 mg/dL, or individuals receiving lipid-lowering medications. Diabetic patients were defined as individuals receiving insulin or oral hypoglycemic agents or those with a glycated hemoglobin (HbA1c) level ≥6.5 %, fasting plasma glucose level ≥126 mg/dL, or non-fasting plasma glucose level ≥200 mg/dL.

Blood samples were drawn before emergent percutaneous coronary intervention on admission. The serum samples were stored at −30 °C until assayed. The serum PUFAs composition, including the levels of EPA, DHA, and AA, was measured by gas–liquid chromatography at a commercially available laboratory (SRL, Tokyo, Japan) [8, 9]. The intra- and inter-assay coefficients of variation for the EPA, DHA, and AA measurements were 1.3 and 3.3 %, 1.5 and 2.2 %, and 1.1 and 2.2 %, respectively. Since n-6 PUFAs, including AA, are considered pro-inflammatory fatty acids, and since the EPA/AA ratio is known to be associated with the incidence of cardiac events, the EPA/AA and DHA/AA ratios were calculated [5]. In addition, other biochemical parameters, including LDL-C, HDL-C, triglycerides, and HbA1c, were also measured. Body mass index was calculated as an index of obesity. The smoking status of the patient was obtained by interviews.

Written informed consent was not required because of the retrospective nature of the investigation. This study protocol was approved by the Tokushima University Hospital Ethics Committee and was conducted in accordance with the Declaration of Helsinki.

### Statistical analyses

The Shapiro-Wilk test was performed to evaluate whether or not the parameters were normally distributed. Non-normally distributed parameters were expressed as median and quartiles. Categorical parameters are expressed as numbers and percentages. After stratification according to age, differences in the clinical characteristics were determined by one-way ANOVA or Chi-square tests. Pearson correlation analysis was performed to determine the association between the age at ACS onset and the EPA/AA or DHA/AA ratio. Multiple regression analysis was used to assess the degrees of association among the variables, with age at ACS onset as the outcome variable. Age, body mass index, and the triglyceride, HDL-C, LDL-C, HbA1c, EPA, DHA, AA, EPA/AA, and DHA/AA levels were natural log transformed for the statistical analyses, because of their non-normal distributions. All statistical analyses were performed using JMP 10 software (SAS, Cary, NC, USA). Statistical significance was defined as *P* < 0.05.

## Results

### Clinical characteristics of subjects

The characteristics of all patients stratified by age are shown in Table 1. There were significant differences in the EPA/AA ratios among the groups. In addition, there were significant differences in the male sex ratio, body mass index, and HDL-C levels, prevalence of current smokers, and prevalence of angiotensin-converting enzyme inhibitors/angiotensin II receptor blocker use, but not in the triglyceride, LDL-C, HbA1c, EPA, DHA, and AA levels, DHA/AA ratio, prevalence of dyslipidemia, hypertension, and diabetes mellitus, and the other administered drugs. Pearson correlation analysis showed that the levels of EPA and DHA were positively associated with the EPA/AA and DHA/AA ratios, respectively, in all patients (Additional file 1: Figure S1).

**Table 1:** Clinical characteristics of all patients stratified by age

Variables	Total	35–50 y	51–74 y	≥75 y	*P*-value
Number of patients	102	11	57	34	–
Male, n (%)	73 (71.6 %)	11 (100 %)	46 (80.7 %)	16 (47.1 %)	<0.001
Age (years)	69 (56–77)	42 (38–49)	64 (56–70)	79 (77–84)	<0.001
Body mass index (kg/m^2^)	24 (22–25)	26 (25–29)*,††	24 (21–26)	22 (20–24)	<0.01
Triglycerides (mg/dL)	111 (67–171)	143 (132–194)	110 (63–177)	95 (66–129)	0.09
HDL-C (mg/dL)	45 (39–59)	42 (34–43)*	47 (40–60)	45 (38–60)	0.04
LDL-C (mg/dL)	114 (92–137)	133 (101–185)	113 (90–132)	117 (84–148)	0.09
HbA1c (%)	5.9 (5.6–6.4)	5.8 (5.4–6.7)	6.0 (5.5–6.6)	5.9 (5.6–6.4)	0.53
Fatty acid concentrations					
EPA (μg/mL)	43 (29–65)	28 (21–55)*,†	43 (34–65)	46 (30–69)	0.12
DHA (μg/mL)	119 (97–149)	102 (94–140)	118 (97–150)	125 (97–163)	0.36
AA (μg/mL)	165 (141–206)	193 (164–234)	167 (152–203)	143 (126–211)	0.09
EPA/AA	0.25 (0.17–0.37)	0.17 (0.07–0.23)*,†	0.26 (0.18–0.37)	0.29 (0.19–0.42)	0.03
DHA/AA	0.75 (0.59–0.96)	0.70 (0.47–0.89)	0.67 (0.56–0.95)	0.83 (0.70–1.00)	0.05
Complications					
Dyslipidemia, n (%)	38 (37 %)	6 (55 %)	22 (39 %)	10 (29 %)	0.31
Hypertension, n (%)	63 (62 %)	5 (45 %)	34 (60 %)	24 (71 %)	0.29
Diabetes mellitus, n (%)	29 (28 %)	2 (18 %)	19 (33 %)	8 (24 %)	0.44
Current smoking, n (%)	44 (43 %)	9 (82 %)	28 (49 %)	7 (21 %)	<0.01
Drugs					
ACEI/ARB, n (%)	27 (26 %)	1 (9 %)	11 (19 %)	15 (44 %)	0.01
β-blockers, n (%)	10 (10 %)	1 (9 %)	4 (7 %)	5 (15 %)	0.49
Calcium channel blockers, n (%)	27 (26 %)	1 (9 %)	15 (26 %)	11 (32 %)	0.31
Statins, n (%)	15 (15 %)	2 (18 %)	11 (19 %)	2 (6 %)	0.20
Aspirin, n (%)	16 (16 %)	2 (18 %)	8 (14 %)	6 (18 %)	0.28

Unless indicated otherwise, data are presented as median and quartiles

*Abbreviations*: *AA* arachidonic acid, *ACEI* angiotensin converting enzyme inhibitors, *ARB* angiotensin II receptor blockers, *DHA* docosahexaenoic acid, *EPA* eicosapentaenoic acid, *HbA1c* glycated hemoglobin, *HDL-C* high-density lipoprotein cholesterol, *LDL-C* low-density lipoprotein cholesterol

*P <0.05 (vs. age 51–74)

†*P* <0.05 (vs. age ≥75)

††*P* <0.01 (vs. age ≥75)

Of note, the patients aged 35–50 were all men. Therefore, we moreover performed subgroup analyses of male patients stratified by age (Additional file 2: Table S1). A significant difference was observed for the EPA/AA ratio, but not DHA/AA.

The female patient characteristics stratified by age are shown (Additional file 2: Table S2).

### Correlation between n-3 PUFAs and age at ACS onset

**Fig. 1:**
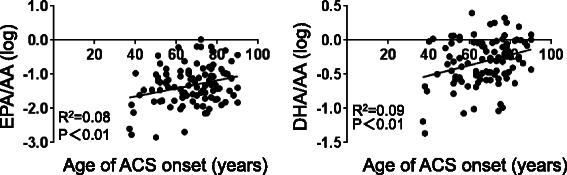
Pearson correlation analyses in all patients between the age at ACS onset and levels of EPA/AA and DHA/AA. *AA* arachidonic acid, *ACS* acute coronary syndrome, *DHA* docosahexaenoic acid, *EPA* eicosapentaenoic acid

The Pearson correlation analysis showed that age at ACS onset was positively associated with the EPA/AA and DHA/AA ratios in all (Fig. 1) and male patients (Additional file 1: Figure S2).

Furthermore, multiple regression analysis in all patients revealed that male sex, and body mass index, prevalence of current smoker, and level of triglycerides were negative, and levels of EPA/AA or DHA/AA were positive contributors to the age at ACS onset (Table 2, Model 1, 2). However, levels of LDL-C, HDL-C, HbA1c, and prevalence of hypertension were statistically excluded. Multiple regression analysis in male patients revealed that levels of EPA/AA or DHA/AA were positive contributors and level of triglycerides was a negative contributor to the age at ACS onset. However, the body mass index, levels of LDL-C, HDL-C, and HbA1c, prevalence of hypertension and current smoker status were statistically excluded (Additional file 2: Table S3, Model 1, 2).

**Table 2:** Multiple regression analysis for determinants of the age of acute coronary syndrome onset

Variables	Coefficient	95 % CI	*P*-value
Model 1			
Male sex	−0.06	−0.10 to −0.02	<0.01
Body mass index	−0.27	−0.50 to −0.05	0.02
Hypertension	0.04	−0.001 to 0.07	0.05
Current smoker	−0.04	−0.08 to 0.001	0.06
LDL-C	−0.04	−0.12 to 0.04	0.28
Triglycerides	−0.08	−0.14 to −0.02	0.01
HDL-C	0.01	−0.13 to 0.16	0.85
HbA1c, %	0.20	−0.03 to 0.44	0.09
EPA/AA	0.07	0.01 to 0.13	0.02
Model 2			
Male sex	−0.06	−0.10 to −0.02	<0.01
Body mass index	−0.24	−0.46 to −0.01	0.04
Hypertension	0.03	−0.001 to 0.07	0.05
Current smoker	−0.04	−0.08 to 0.01	0.04
LDL-C	−0.04	−0.11 to 0.04	0.31
Triglycerides	−0.09	−0.15 to −0.03	<0.01
HDL-C	0.05	−0.10 to 0.19	0.53
HbA1c, %	0.18	−0.05 to 0.41	0.12
DHA/AA	0.15	−0.05 to 0.25	<0.01

Model 1, R^2^ = 0.37; *P* <0.001

Model 2, R^2^ = 0.39; *P* <0.001

*Abbreviations*: *AA* arachidonic acid, *CI* confidence interval, *DHA* docosahexaenoic acid; *EPA* eicosapentaenoic acid, *HbA1c* glycated hemoglobin, *HDL-C* high-density lipoprotein cholesterol, *LDL-C* low-density lipoprotein cholesterol

## Discussion

Herein, we showed that the EPA/AA ratio was the lowest in early-onset ACS patients and that low levels of EPA/AA and DHA/AA were independent contributors of early onset of ACS.

Although patients with CAD typically become symptomatic after age 40, autopsy studies have demonstrated that coronary atherosclerosis can begins as early as 20 years of age [10–13], and such atherosclerotic changes in the coronary arteries have also been observed in Japanese youths, whose incidence and mortality from CAD are reportedly the lowest in all industrialized countries [14].

Despite recent advances in the identification of risk factors, the occurrence of CAD has continued to increase worldwide, and despite the decrease in cardiovascular mortality due to statin treatment, two out of three patients still experience cardiovascular events [15]. In addition, the AMI hospitalization rate for relatively young people has remained stable over the past decade [16–18], which may be largely due to the insufficient control of the various CAD risk factors [1]. Several risk factors for early-onset AMI have been reported; classical risk factors include serum cholesterol levels, systolic blood pressure, and cigarette smoking [19], while non-classical risk factors include vasospastic tendencies, thrombophilic conditions, and a history of Kawasaki disease [20, 21]. However, there are currently no universal guidelines on the prevention of CAD in young people, in part because of insufficient evidence on coronary disease prevention in adults younger than 40 years [19]. Therefore, sufficient control of classical CAD risk factors and identification of residual risk factors for CAD are needed.

It has been reported that reduced serum levels of n-3 PUFA, especially EPA, are associated with an increased incidence of cardiovascular events and mortality [3–5], and the JELIS study, a large clinical trial, showed effects of EPA on reducing cardiovascular events in hypercholesterolemic patients [22]. However, it should be noted that the levels of EPA (95 μg/mL) and DHA (170 μg/mL) in the hyperlipidemic patients without ACS enrolled in the JELIS trial were higher than those in the ACS patients in our study (EPA, 43 μg/mL; DHA, 119 μg/mL) [23]. Furthermore, the Japanese National Health and Nutrition Survey demonstrated that the type of nutrition has changed from Japanese traditional style to western style; in particular, fish intake has been decreasing, especially in people aged 30–49 and 40–49 years (http://www0.nih.go.jp/eiken/english/research/program_epidemiology.html), suggesting that low levels of n-3 PUFAs could lead to CAD prevention in young people.

In the study, all patients aged 50 or under were men. It has been well established that male gender is a risk factor of CAD [24, 25], and previous studies have shown that men with ACS are more likely to have ruptured plaques, whereas women are more likely to present with plaque erosion, suggesting that the mechanism of ACS development in men and women differs [24]. For example, it has been speculated that estrogen might contribute to delaying the plaque development, stabilizing the existing plaques, and preventing plaque rupture in women [25].

On the other hand, the underlying mechanisms responsible for the favorable effects of n-3 PUFAs remain partly unknown. n-3 PUFAs have been demonstrated to induce stability of vulnerable plaques, which is considered to contribute to the protection against acute cardiovascular events, and it has been shown that administration of fish oil increased the proportions of n-3 PUFAs in carotid plaque lipids and increased the proportions of well-formed fibrous caps, rather than thin inflamed caps, as compared with a placebo or sunflower oil [26]. In addition, a recent study showed that administration of EPA increased the fibrous cap thickness of coronary atherosclerotic plaques [27].

Moreover, several studies have shown that long-chain n-3 PUFAs can decrease the expressions of intercellular adhesion molecule-1 and vascular cell adhesion molecule-1 on the surface of endothelial cells [28] and monocytes [29]. Therefore, n-3 PUFA-induced decreases in adhesion molecule expression on endothelial cells and/or the monocyte/macrophage itself, might reduce migration of monocytes/macrophages into the plaque [30, 31]. Further, chylomicron remnant-like particles enriched in n-3 PUFA are taken up more slowly by macrophages than those enriched in saturated or monounsaturated fatty acids, which might also be involved in attenuation of plaque formation [32], and it has been shown that administration of n-3 PUFA reduced the matrix metalloproteinase-7, −9 and −12 expressions [33]. These gene products are proteases capable of degrading extracellular matrix proteins, consequently leading to plaque rupture [33]. Accordingly, reduced expression of matrix metalloproteinases by n-3 PUFAs may also contribute to plaque instability, leading to suppression of ACS.

Although some meta-analyses and clinical trials have shown beneficial effects of n-3 PUFAs on cardiovascular events [22, 34], a previous systematic review conversely showed no definite effects of n-3 PUFAs on cardiovascular events [35]. In addition, some theoretical unfavorable effects have been reported, including one study showing that intake of dietary PUFAs such as EPA and DHA increased lipid peroxidation, which may in turn enhance oxidative stress and cancel the preferable effects of n-3 PUFAs [36]. This unfavorable effect of n-3 PUFAs may explain the inconsistent clinical results and contribute to the finding that the characteristics of patients who received beneficial effects of PUFAs differ among studies [37, 38].

The present study had several limitations. This was a retrospective study—with a relatively small sample size—that was performed in a single-center located in a costal provincial city. Larger clinical cohort studies are needed to confirm our results and to clarify the effects of n-3 PUFAs on early-onset ACS.

## Conclusions

We here demonstrated that decreased EPA/AA and DHA/AA ratios may be risk factors for early onset of ACS, suggesting that reduced EPA/AA and DHA/AA may represent targets for preventing ACS in Japanese young people.

## Additional files

Additional file 1: **Figure S1.** Pearson correlation analysis showed that the levels EPA and DHA were positively associated with the EPA/AA and DHA/AA ratios, respectively, in all patients. AA, arachidonic acid; DHA, docosahexaenoic acid; EPA, eicosapentaenoic acid. **Figure S2.** Pearson correlation analyses in male patients between the age at ACS onset and levels of EPA/AA and DHA/AA. AA, arachidonic acid; ACS, acute coronary syndrome; DHA, docosahexaenoic acid; EPA, eicosapentaenoic acid. (PPTX 101 kb) Additional file 2: **Table S1.** Clinical characteristics of the male patients stratified by age. **Table S2.** Clinical characteristics of the female patients stratified by age. **Table S3.** Multiple regression analysis for contributors of the age of acute coronary syndrome onset in male patients. (DOCX 25 kb)

## Abbreviations

ACS: acute coronary syndrome
AMI: acute myocardial infarction
CAD: coronary artery disease
PUFAs: polyunsaturated fatty acids
EPA: eicosapentaenoic acid
DHA: docosahexaenoic acid
AA: arachidonic acid
LDL-C: low-density lipoprotein cholesterol
HDL-C: high-density lipoprotein cholesterol
HbA1c: glycated hemoglobin
