# Erratum to: ‘Reduced ratio of eicosapentaenoic acid and docosahexaenoic acid to arachidonic acid is associated with early onset of acute coronary syndrome’

**DOI:** 10.1186/s12937-015-0112-2

**Published:** 2015-12-01

**Authors:** Shusuke Yagi, Ken-ichi Aihara, Daiju Fukuda, Akira Takashima, Mika Bando, Tomoya Hara, Sachiko Nishimoto, Takayuki Ise, Kenya Kusunose, Koji Yamaguchi, Takeshi Tobiume, Takashi Iwase, Hirotsugu Yamada, Takeshi Soeki, Tetsuzo Wakatsuki, Michio Shimabukuro, Masashi Akaike, Masataka Sata

**Affiliations:** Department of Cardiovascular Medicine, Institute of Biomedical Sciences, Tokushima University Graduate School, 3-18-15, Kuramoto, Tokushima 770-8503 Japan; Department of Hematology, Endocrinology and Metabolism, Institute of Biomedical Sciences, Tokushima University Graduate School, 3-18-15, Kuramoto, Tokushima 770-8503 Japan; Department of Nutrition and Metabolism, Institute of Biomedical Sciences, Tokushima University Graduate School, 3-18-15, Kuramoto, Tokushima 770-8503 Japan; Department of Cardio-Diabetes Medicine, Institute of Biomedical Sciences, Tokushima University Graduate School, 3-18-15, Kuramoto, Tokushima 770-8503 Japan; Department of Medical Education, Institute of Biomedical Sciences, Tokushima University Graduate School, 3-18-15, Kuramoto, Tokushima 770-8503 Japan

After the publication of [1] it came to the authors’ attention that the article’s Table 2, model 2 was missing a bottom row for DHA/AA. The article has now been updated with this row and it is also included here.

**Table 2:** Multiple regression analysis for determinants of the age of acute coronary syndrome onset

Variables	Coefficient	95 % CI	P-value
Model 1			
Male sex	−0.06	−0.10 to −0.02	<0.01
Body mass index	−0.27	−0.50 to −0.05	0.02
Hypertension	0.04	−0.001 to 0.07	0.05
Current smoker	−0.04	−0.08 to 0.001	0.06
LDL-C	−0.04	−0.12 to 0.04	0.28
Triglycerides	−0.08	−0.14 to −0.02	0.01
HDL-C	0.01	−0.13 to 0.16	0.85
HbA1c, %	0.20	−0.03 to 0.44	0.09
EPA/AA	0.07	0.01 to 0.13	0.02
Model 2			
Male sex	−0.06	−0.10 to −0.02	<0.01
Body mass index	−0.24	−0.46 to −0.01	0.04
Hypertension	0.03	−0.001 to 0.07	0.05
Current smoker	−0.04	−0.08 to 0.01	0.04
LDL-C	−0.04	−0.11 to 0.04	0.31
Triglycerides	−0.09	−0.15 to −0.03	<0.01
HDL-C	0.05	−0.10 to 0.19	0.53
HbA1c, %	0.18	−0.05 to 0.41	0.12
DHA/AA	0.15	0.05 to 0.25	<0.01

R^2^ = 0.37; *P <* 0.001

Model 1, R ^2^ = 0.37; *P <* 0.001

Model 2, R ^2^ = 0.39; *P <* 0.001

*Abbreviations*: *AA* arachidonic acid, *CI* confidence interval, *DHA* docosahexaenoic acid, *EPA* eicosapentaenoic acid, *HbA1c* glycated hemoglobin, *HDL-C* high-density lipoprotein cholesterol, *LDL-C* low-density lipoprotein cholesterol
