# Supplementary material for: Reduced ratio of eicosapentaenoic acid and docosahexaenoic acid to arachidonic acid is associated with early onset of acute coronary syndrome
Source: Nutr J. 2015 Oct 29;14:111. doi: 10.1186/s12937-015-0102-4 (PMC4627394; doi:10.1186/s12937-015-0102-4)
Supplement: Additional file 1: — Figure S1. Pearson correlation analysis showed that the levels EPA and DHA were positively associated with the EPA/AA and DHA/AA ratios, respectively, in all patients. AA, arachidonic acid; DHA, docosahexaenoic acid; EPA, eicosapentaenoic acid. Figure S2. Pearson correlation analyses in male patients between the age at ACS onset and levels of EPA/AA and DHA/AA. AA, arachidonic acid; ACS, acute coronary syndrome; DHA, docosahexaenoic acid; EPA, eicosapentaenoic acid. (PPTX 101 kb) [file 12937_2015_102_MOESM1_ESM.pptx]

## Slide 1
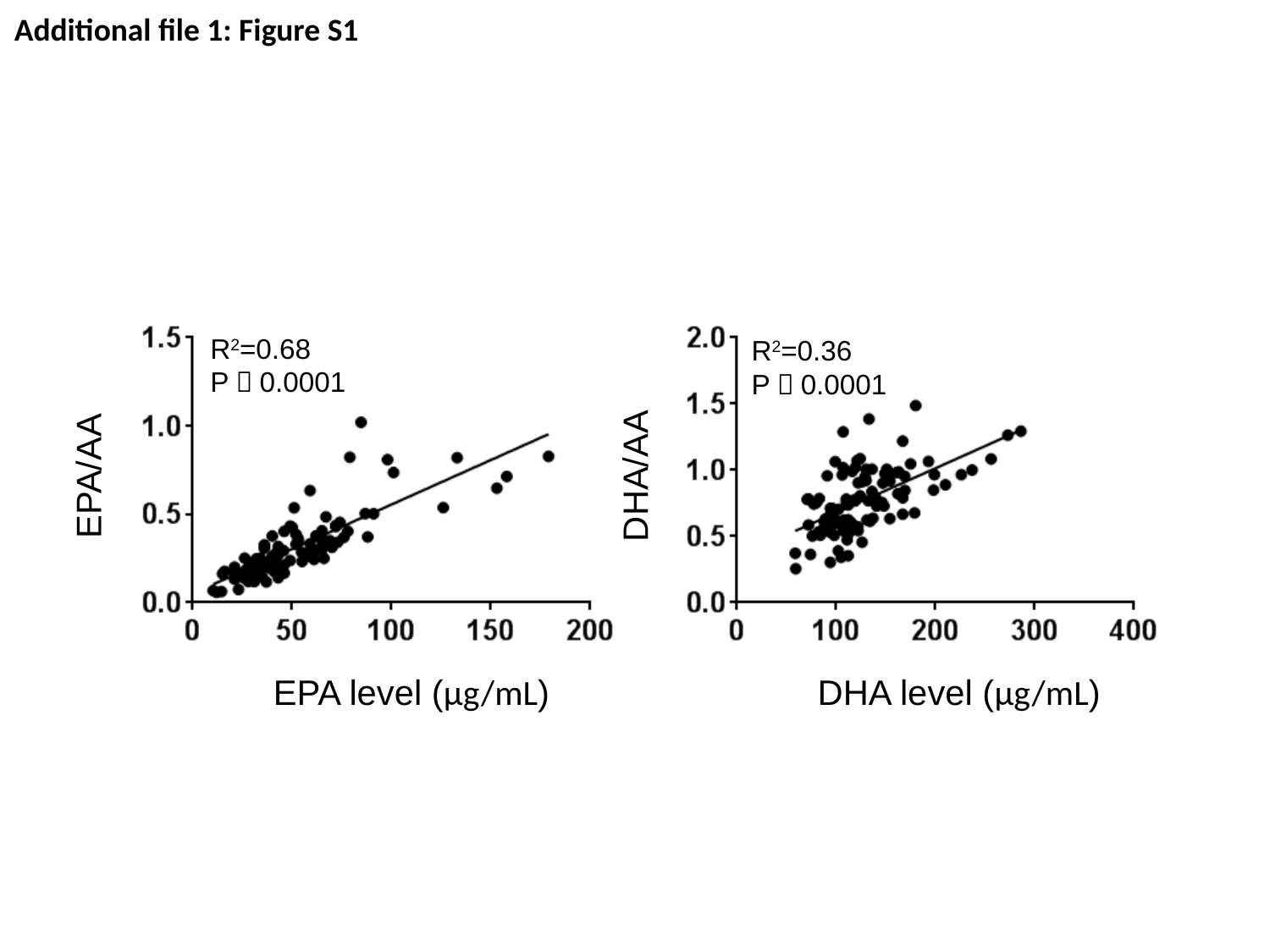

Additional file 1: Figure S1
R2=0.68
P＜0.0001
R2=0.36
P＜0.0001
EPA/AA
DHA/AA
EPA level (μg/mL)
DHA level (μg/mL)

## Slide 2
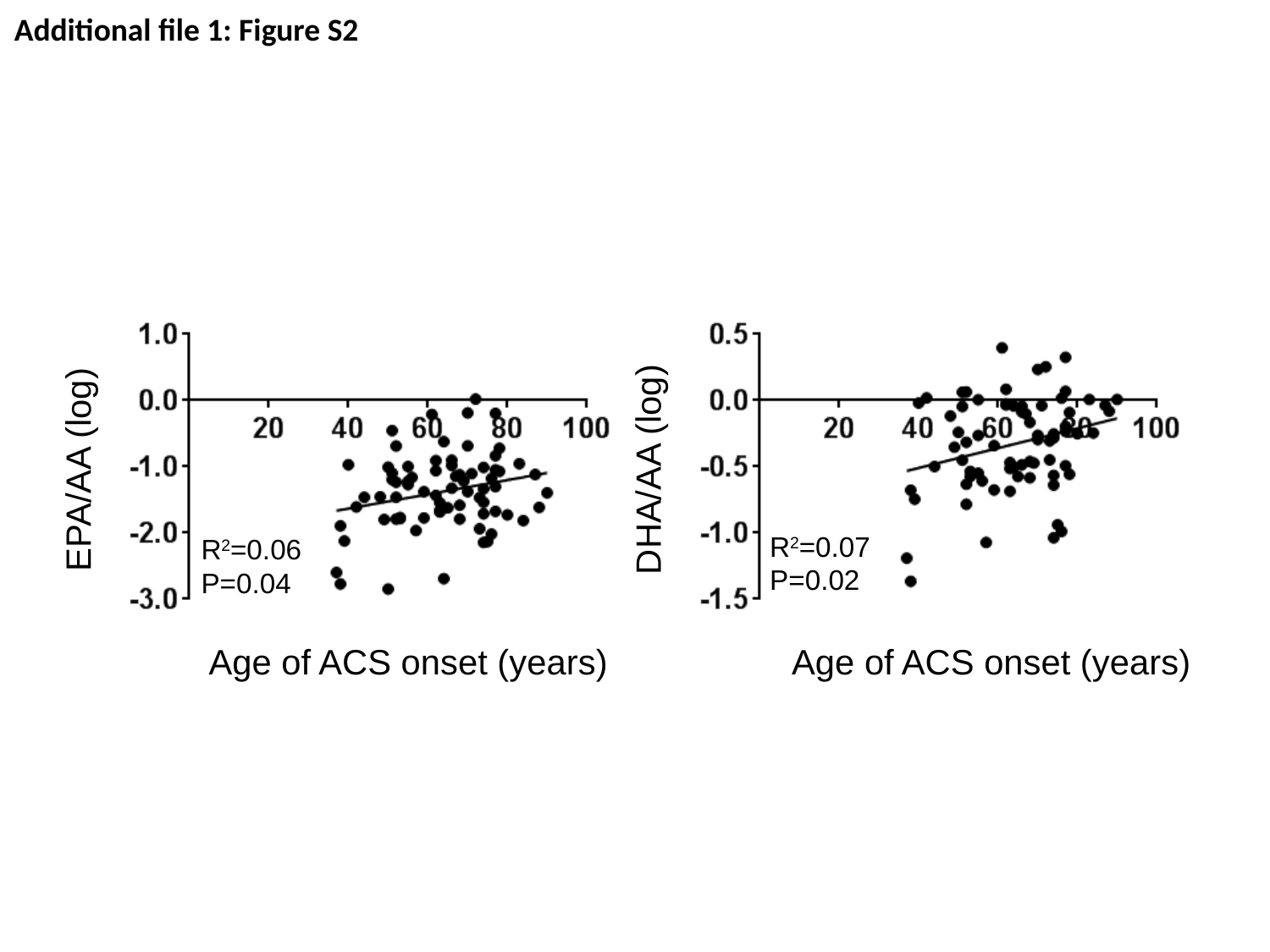

Additional file 1: Figure S2
EPA/AA (log)
DHA/AA (log)
R2=0.07
P=0.02
R2=0.06
P=0.04
Age of ACS onset (years)
Age of ACS onset (years)
