# Supplementary material for: Reduced ratio of eicosapentaenoic acid and docosahexaenoic acid to arachidonic acid is associated with early onset of acute coronary syndrome
Source: Nutr J. 2015 Oct 29;14:111. doi: 10.1186/s12937-015-0102-4 (PMC4627394; doi:10.1186/s12937-015-0102-4)
Supplement: Additional file 2: — Table S1. Clinical characteristics of the male patients stratified by age. Table S2. Clinical characteristics of the female patients stratified by age. Table S3. Multiple regression analysis for contributors of the age of acute coronary syndrome onset in male patients. (DOCX 25 kb) [file 12937_2015_102_MOESM2_ESM.docx]

**Additional file 2: Table S1**

**Clinical characteristics of the male patients stratified by age**

| **Variables** | **Total** | **35-50 y** | **51-74 y** | **≥75 y** | **P-value** |
| --- | --- | --- | --- | --- | --- |
| Number of patients | 73 | 11 | 46 | 16 |  |
| Age (years) | 65 (52-74) | 42 (38-49) | 63 (55-70) | 78 (77-83) | <0.001 |
| Body mass index (kg/m^2^) | 24(21-26) | 26 (25-29)*† | 23 (21-25) | 23 (20-25) | <0.01 |
| Triglycerides (mg/dL) | 110 (65-172) | 143 (132-194) | 109 (60-176) | 76 (65-126) | 0.08 |
| HDL-C (mg/dL) | 43 (37-55) | 42 (32-43) | 47 (40-59) | 43 (36-53) | 0.08 |
| LDL-C (mg/dL) | 113 (92-134) | 133 (101-185)† | 113 (92-131) | 94 (78-120) | 0.03 |
| HbA1c (%) | 5.8 (5.6-6.3) | 5.8 (5.4-6.7) | 5.9 (5.5-6.3) | 5.7 (5.6-6.2) | 0.85 |
| Fatty acid concentrations |  |  |  |  |  |
| EPA (μg/mL) | 43 (28-60) | 28 (21-55) | 46 (33-62) | 36 (27-64) | 0.10 |
| DHA (μg/mL) | 113 (95-136) | 102 (94-140) | 115 (95-148) | 121 (91-132) | 0.64 |
| AA (μg/mL) | 164 (136-206) | 193 (164-234)† | 164 (149-203) | 136 (110-187) | 0.04 |
| EPA/AA | 0.25 (0.17-0.35) | 0.16 (0.07-0.23)**† | 0.27 (0.18-0.36) | 0.28 (0.17-0.37) | <0.01 |
| DHA/AA | 0.75 (0.57-0.96) | 0.70 (0.47-0.89) | 0.72 (0.56-0.95) | 0.86 (0.65-1.00) | 0.22 |
| Complications |  |  |  |  |  |
| Dyslipidemia, n (%) | 26 (36%) | 6 (55%) | 17 (37%) | 3 (19%) | 0.15 |
| Hypertension, n (%) | 42 (58%) | 5 (46%) | 27 (59%) | 10 (63%) | 0.66 |
| Diabetes mellitus, n (%) | 19 (26%) | 2 (18%) | 13 (28%) | 4 (25%) | 0.79 |
| Current smoking, n (%) | 40 (55%) | 9 (82%) | 26 (57%) | 5 (31%) | 0.03 |
| Drugs |  |  |  |  |  |
| ACEI/ARB, n (%) | 17 (23%) | 1 (9%) | 9 (20%) | 7 (44%) | 0.08 |
| β-blockers, n (%) | 6 (8%) | 1 (9%) | 4 (9%) | 1 (6%) | 0.94 |
| Calcium channel blockers, n (%) | 15 (21%) | 1 (9%) | 10 (22%) | 4 (25%) | 0.53 |
| Statins, n (%) | 10 (14%) | 2 (18%) | 7 (15%) | 1 (6%) | 0.60 |
| Aspirin, n (%) | 11 (15%) | 2 (18%) | 7 (15%) | 1 (6%) | 0.19 |

Unless indicated otherwise, data are presented as median and quartiles.

Abbreviations: AA, arachidonic acid; ACEI, angiotensin converting enzyme inhibitors; ARB, angiotensin II receptor blockers; DHA, docosahexaenoic acid; EPA, eicosapentaenoic acid; HbA1c, glycated hemoglobin; HDL-C, high-density lipoprotein cholesterol; LDL-C, low-density lipoprotein cholesterol.

*P<0.05 (vs. age 51-74), †P<0.05 (vs. age ≥75), **P<0.01 (vs. age 51-74)

**Additional file 2: Table S2**

**Clinical characteristics of the female patients stratified by age**

| **Variables** | **Total** | **51-74 y** | **≥75 y** | **P-value** |
| --- | --- | --- | --- | --- |
| Number of patients | 29 | 11 | 18 |  |
| Age (years) | 78 (70-83) | 66 (62-72) | 81 (78-85) | <0.001 |
| Body mass index (kg/m^2^) | 22 (21-25) | 24 (22-27) | 22 (20-24) | 0.04 |
| Triglycerides (mg/dL) | 113 (73-177) | 189 (147-210) | 103 (70-133) | 0.62 |
| HDL-C (mg/dL) | 47 (43-64) | 47 (43-70) | 46 (43-63) | 0.84 |
| LDL-C (mg/dL) | 127 (92-146) | 100 (85-137) | 129 (96-154) | 0.13 |
| HbA1c (%) | 6.0 (5.6-6.5) | 6.1 (5.5-7.5) | 6.0 (5.6-6.4) | 0.10 |
| Fatty acid concentrations |  |  |  |  |
| EPA (μg/mL) | 50 (34-76) | 43 (35-72) | 51 (33-91) | 0.57 |
| DHA (μg/mL) | 136 (107-171) | 136 (108-163) | 134 (97-205) | 0.35 |
| AA (μg/mL) | 173 (148-227) | 192 (167-203) | 156 (136-233) | 0.21 |
| EPA/AA | 0.27 (0.20-0.43) | 0.21 (0.14-0.43) | 0.31 (0.22-0.47) | 0.44 |
| DHA/AA | 0.75 (0.63-0.97) | 0.63 (0.55-0.83) | 0.82 (0.70-0.99) | 0.04 |
| Complications |  |  |  |  |
| Dyslipidemia, n (%) | 12 (41%) | 5 (44%) | 7 (39%) | 0.72 |
| Hypertension, n (%) | 21 (72%) | 7 (64%) | 14 (78%) | 0.41 |
| Diabetes mellitus, n (%) | 10 (34%) | 6 (55%) | 4 (22%) | 0.08 |
| Current smoking, n (%) | 4 (14%) | 2 (18%) | 2 (11%) | 0.59 |
| Drugs |  |  |  |  |
| ACEI/ARB, n (%) | 10 (34%) | 2 (18%) | 8 (44%) | 0.15 |
| β-blockers, n (%) | 4 (14%) | 0 (0%) | 4 (22%) | 0.04 |
| Calcium channel blockers, n (%) | 12 (41%) | 5 (45%) | 7 (39%) | 0.73 |
| Statins, n (%) | 5 (17%) | 4(36%) | 1 (0.1%) | 0.03 |
| Aspirin, n (%) | 5 (17%) | 1 (0.1%) | 4 (22%) | 0.63 |

Unless indicated otherwise, data are presented as median and quartiles.

Abbreviations: AA, arachidonic acid; ACEI, angiotensin converting enzyme inhibitors; ARB, angiotensin II receptor blockers; DHA, docosahexaenoic acid; EPA, eicosapentaenoic acid; HbA1c, glycated hemoglobin; HDL-C, high-density lipoprotein cholesterol; LDL-C, low-density lipoprotein cholesterol.

**Additional file 2: Table S3**

**Multiple regression analysis for contributors of the age of acute coronary syndrome onset in male patients**

**Model 1**

| **Variables** | **Coefficient** | **95% CI** | **P-value** |
| --- | --- | --- | --- |
| Body mass index | −0.21 | −0.56 to 0.12 | 0.21 |
| Hypertension | 0.04 | −0.01 to 0.08 | 0.12 |
| Current smoker | -0.04 | −0.08 to 0.01 | 0.15 |
| LDL-C | −0.06 | −0.15 to 0.03 | 0.18 |
| Triglycerides | −0.08 | −0.16 to 0.01 | 0.06 |
| HDL-C | 0.07 | −0.12 to 0.26 | 0.50 |
| HbA1c | 0.27 | −0.02 to 0.57 | 0.07 |
| EPA/AA | 0.08 | 0.01 to 0.16 | 0.04 |

R^2^ = 0.35; P < 0.001

**Model 2**

| **Variables** | **Coefficient** | **95% CI** | **P-value** |
| --- | --- | --- | --- |
| Body mass index | −0.17 | −0.56 to 0.12 | 0.32 |
| Hypertension | 0.03 | −0.01 to 0.08 | 0.15 |
| Current smoker | −0.04 | −0.08 to 0.01 | 0.10 |
| LDL-C | −0.06 | −0.15 to 0.03 | 0.20 |
| Triglycerides | −0.08 | −0.16 to −0.01 | 0.04 |
| HDL-C | 0.11 | −0.09 to 0.30 | 0.27 |
| HbA1c | 0.23 | −0.07 to 0.52 | 0.13 |
| DHA/AA | 0.16 | 0.03 to 0.28 | 0.02 |

R^2^ = 0.37; P < 0.001

Abbreviations: AA, arachidonic acid; CI, confidence interval; DHA, docosahexaenoic acid; EPA, eicosapentaenoic acid; HbA1c, glycated hemoglobin; HDL-C, high-density lipoprotein cholesterol; LDL-C, low-density lipoprotein cholesterol.
